# Gut microbiome dynamics and functional shifts in healthy aging: insights from a metagenomic study

**DOI:** 10.3389/fmicb.2025.1629811

**Published:** 2025-09-18

**Authors:** Xu Ai, Chenchen Huang, Qiongrong Liu, Rui Duan, Xu Ma, Linzi Li, Zitan Shu, Yuanxin Miao, Hexiao Shen, Yongling Lv, Zhiwei Jiang, Hong Luo, Zhou Long

**Affiliations:** ^1^Jingmen Central Hospital, Jingmen Central Hospital Affiliated to Jingchu University of Technology, Jingmen, Hubei, China; ^2^Hubei Clinical Medical Research Center for Functional Colorectal Diseases, Jingmen, Hubei, China; ^3^College of Food and Biology, Jingchu University of Technology, Jingmen, Hubei, China; ^4^Maintainbiotech Ltd., Wuhan, Hubei, China; ^5^College of Life Science and Technology, Huazhong University of Science and Technology, Wuhan, China

**Keywords:** aging, microbial diversity, functional pathways, gut, microbiota

## Abstract

**Introduction:**

Population aging represents a significant challenge in contemporary society. The gut microbiome plays a critical role in maintaining host health and physiological functions, and its alterations with advancing age are closely associated with the process of healthy aging.

**Methods:**

This study conducted a comprehensive analysis of the gut microbiome in hundred healthy elderly individuals (aged ≥60) residing in Changshou Town, Zhongxiang City, Hubei Province, utilizing metagenomic sequencing technology. The primary objective was to investigate the changes in the gut microbiome and its potential functions during the latter stages of life. Participants were categorized into three distinct age groups: the Young-Old group (YO, ages 60-74), the Middle-Old group (MO, ages 75-89), and the Long-Lived Old group (LO, ages 90-99).

**Results:**

The findings indicate that the diversity of the gut microbiome tends to diminish with age. However, a significant reversal was observed among healthy longevity elderly individuals. Our analysis specifically focused on the trends in the alterations of gut microbiome species and their potential functions as age increases, revealing that the changes in major differential functions closely align with the trends in major differential species, demonstrating a strong positive correlation. The YO group exhibited a more diverse array of differential microbial characteristics and functional traits. Notably, there was a significant enrichment of *Bacteroides stercoris* in the YO group, which displayed a continuous decline with age, alongside a marked enrichment of pathways associated with xenobiotic biodegradation and metabolism. Furthermore, species significantly linked to aging-related pathways, such as oxidative phosphorylation, were identified through species functional correlation analysis. Specifically, *Collinsella bouchesdurhonensis* and *Prevotella stercorea* were enriched in the LO and YO groups, respectively. In total, we successfully obtained two hundred and thirty eight high-quality bins through metagenomic assembly, which included the identification of four species with 100% completeness, as well as the genomic information of the *Methanobrevibacter smithii A* across all groups.

**Discussion:**

This study characterizes the age-associated trends in gut microbiome composition and function during later-life healthy aging, providing exploratory insights that may inform future microecological intervention strategies, pending validation in longitudinal studies.

## 1 Introduction

Population aging represents a significant social challenge that warrants attention in contemporary society. As individuals progress through the aging process, the human gut microbiota experiences gradual alterations (Odamaki et al., 2016; Fu et al., 2023). The human gut microbiota is a complex and dynamically evolving community of microorganisms residing within the gastrointestinal tract, which plays a critical role in maintaining physiological functions and overall host health (Adak and Khan, 2019; Gomaa, 2020). This microbiome is not only integral to essential physiological processes such as digestion, metabolism, and immune regulation, but it also possesses the capacity to influence brain function through the gut-brain axis (Koh and Bäckhed, 2020; Liu et al., 2022; Mitra et al., 2023). Aging, illness, and mortality are inevitable aspects of life, and numerous studies conducted in recent years have highlighted the crucial role of the gut microbiota in various diseases (Fan and Pedersen, 2021; Afzaal et al., 2022; Hou et al., 2022). However, our understanding of its potential contributions to the aging process and the promotion of healthy aging remains limited.

The relationship between the gut microbiota and aging is characterized by a bidirectional interaction (Nagpal et al., 2018). Aging serves as a significant intrinsic factor that influences both the composition and functionality of the gut microbiota. This relationship is closely associated with an increased vulnerability to diseases, which arises from the gradual decline in physiological functions linked to the aging process, as well as the administration of therapeutic agents such as antibiotics (Wu et al., 2021; Ling et al., 2022; Kelso, 2024). Typically, the diversity of the gut microbiota stabilizes following the onset of adulthood. However, it tends to diminish with advancing age, a multifaceted biological phenomenon (Kim and Jazwinski, 2018). Furthermore, alterations in this diversity are frequently accompanied by a rise in opportunistic pathogens and a reduction in beneficial bacteria. For example, the populations of beneficial bacteria, such as *Bifidobacteria* and *Lactobacilli*, often decrease with age, while opportunistic pathogens, including *Enterobacter, Clostridium perfringens*, and *Clostridium difficile*, may proliferate (Claesson et al., 2011; Odamaki et al., 2016; Mitchell et al., 2017; Ling et al., 2022). This dysbiosis, which is characterized by inflammation and metabolic disturbances, is often closely linked to chronic conditions such as inflammatory bowel disease, type 2 diabetes, cardiovascular diseases, and neurodegenerative disorders (Hou et al., 2022). These chronic diseases, in turn, exacerbate age-related health challenges and accelerate the aging process. A more stable and diverse gut microbiota is conducive to promoting healthy aging (Kumar et al., 2016; Ragonnaud and Biragyn, 2021). Strategies such as supplementation with probiotics, prebiotics, synbiotics, psychobiotics, and antioxidants, along with dietary modifications and physical exercise, are considered potential interventions to mitigate aging by modifying the gut microbiota (Juárez-Fernández et al., 2020; Du et al., 2021; Donati Zeppa et al., 2022; Barone et al., 2022). Additionally, research indicates that the core gut microbiota of older adults differs from that of younger individuals, with the gut microbiota composition in individuals over 60 years exhibiting greater variability (Claesson et al., 2011; Ghosh et al., 2020). Notably, at extreme ages, the distinctive gut microbiota structure observed in centenarians suggests that a more “youthful” and “diverse” gut microbiota configuration is advantageous for health and longevity (Pang et al., 2023).

Current research has demonstrated a significant interaction between the gut microbiota and age. However, the acquisition of biological samples from healthy elderly populations presents considerable challenges due to the high prevalence of various age-related diseases among older adults. In contrast to studies focusing on gut microbiota in children, adolescents, and middle-aged healthy populations, there exists a notable deficiency in relevant research data pertaining to healthy elderly individuals. Consequently, this study aims to investigate the gut microbiota of healthy elderly populations across different age stages in the latter half of life. This study employed high-throughput metagenomic sequencing to investigate differences in gut microbiota across distinct aging stages. Moreover, it reconstructed MAGs and explored their associations with functional pathways, uncovering core microbiota and their metabolic potential relevant to healthy aging—an aspect not deeply addressed in prior research. Furthermore, to find healthy elderly individuals, we have selected the elderly population in Changshou Town, Zhongxiang City, Hubei Province, as the subjects of our research. This region is recognized as a hometown of longevity, providing valuable resources for studying the role of gut microbiota in healthy aging and longevity.

This study aimed to infer the potential evolution of intestinal microecology with age by conducting a cross-sectional analysis of healthy elderly individuals across various age brackets. While the study design was not longitudinal, the presence of multiple age cohorts offered insights into the progression of intestinal micro ecology in the later stages of life. By identifying microbial characteristics and functional changes linked to various age stages within the elderly population, we hope to discover potential targets for interventions that promote healthy aging and extend lifespan. The findings of this study will enhance the broader understanding of the role of the gut microbiota in facilitating healthy aging. Particularly in the context of China's rapidly aging population, this research presents potential opportunities for the development of microbiome-based intervention strategies aimed at improving the health of older adults.

## 2 Materials and methods

### 2.1 Ethical statement

This cohort study received approval from the Ethics Committee of the Central Hospital of Jingmen City, Hubei Province (Accession number: 202302229). Informed consent was obtained from all participants in accordance with the principles outlined in the Declaration of Helsinki.

### 2.2 Study cohort and sample collection

Changshou Town, situated in Zhongxiang City, Hubei Province, served as the recruitment site for a total of 100 participants in this study. The participants were required to meet the following inclusion criteria: (1) born in Changshou Town; (2) continuously resided in the Changshou Town area for at least 5 years; (3) aged ≥60 years. Additionally, for the purpose of metagenomic analysis of fecal samples, individuals who had received antibiotic or microbial agent treatment within the 3 months preceding sample collection were excluded, as were those with severe medical conditions (including cancer, diabetes, cardiovascular diseases, and autoimmune diseases), a family history of gastrointestinal diseases, or a history of intestinal surgery. Fecal samples were collected by participants at their homes using sterile fecal collection devices and were promptly transported to the hospital on dry ice, where they were stored at−80 °C until DNA extraction and analysis. This study employed commonly accepted gerontological age groupings: 60–74 years (young-old, YO), 75–89 years (Middle-Old, MO), and 90–99 years (Long-Lived Old, LO), to represent distinct stages in the later life spectrum.

### 2.3 Metagenomic DNA extraction, library construction, and sequencing

In this study, total DNA was extracted from collected fecal samples utilizing the HiPure Stool DNA Kit (Magen, China), in accordance with the manufacturer's instructions. The concentration of DNA was quantified using a Qubit 4.0 (Thermo Fisher Scientific, U.S.A.), and the integrity of the DNA was evaluated through 1.5% agarose gel electrophoresis. Subsequently, a paired-end library with an insert size of 300 bp was constructed following the guidelines provided by the TrueSeq Nano DNA Library Prep Kit (Illumina, U.S.A.). Quality control of the library was conducted using the Agilent 2100 Bioanalyzer (Agilent Technologies, U.S.A.). Ultimately, the qualified library was sequenced on the Illumina Novaseq 6000 platform (Illumina, U.S.A.) to produce 2 × 150 bp reads.

### 2.4 Quality control and assembly of metagenomic data

The initial assessment of the raw sequencing reads was conducted for quality evaluation utilizing FastQC v0.11.8 (Andrews, 2010). Following this, the reads underwent processing with Trimmomatic v0.39 (Bolger et al., 2014). Cleaned reads were aligned to eliminate host sequences through the application of Bowtie2 v2.4.4 (Langmead and Salzberg, 2012) as part of the KneadData v0.10.0 workflow (https://huttenhower.sph.harvard.edu/kneaddata/). Subsequently, the filtered reads were subjected to *de novo* assembly using MEGAHIT v1.2.9 (Li et al., 2015), with a minimum output contig length established at 500 base pairs.

### 2.5 Taxonomic and functional annotation

Coding sequences (CDS) were predicted from the assembled contigs utilizing MetaGeneMark v3.38 (Zhu et al., 2010), with the exclusion of sequences shorter than 100 bp. The predicted CDS were subsequently clustered using CD-HIT v4.8.1 (Li and Godzik, 2006), applying thresholds for coverage greater than 90% and identity exceeding 95% to minimize redundancy. The resulting non-redundant gene set was then compared against the NCBI NR protein database using DIAMOND v2.0.11 (Buchfink et al., 2015), with alignment results selected based on an e-value threshold of ≤ 10^−5^.

### 2.6 Construction and annotation of metagenome assembled genomes (MAGs)

Metagenomic binning was conducted on clean reads from 100 samples, focusing on contigs longer than 1000 base pairs. This process utilized a combination of three algorithms: MetaBAT2 v2.12.1 (Kang et al., 2019), MaxBin2 v2.2.6 (Wu et al., 2016), and CONCOCT v1.0.0 (Alneberg et al., 2013), all within the MetaWRAP framework v1.3.2 (Uritskiy et al., 2018). High-quality MAGs are defined as those exhibiting a completion rate greater than 90% and a contamination level below 5%. Medium-quality MAGs were characterized by a completion rate exceeding 50% and a contamination level under 10%. The integrity and contamination levels of each MAG were evaluated using CheckM v1.0.12 (Parks et al., 2015), and redundant MAGs were eliminated using dRep v3.4.5 (Olm et al., 2017) based on a standard of 95% average nucleotide identity (ANI). Finally, taxonomic annotation of the MAGs was performed utilizing GTDB-Tk v2.3.2, referencing the Genome Taxonomy Database (GTDB r214, https://gtdb.ecogenomic.org/stats/r214).

### 2.7 Statistical analysis

To conduct a comprehensive assessment of the structure and function of the gut microbiome, we employed a variety of statistical analyses. Initially, we calculated the alpha diversity index for each sample utilizing QIIME 2 (Bolyen et al., 2019) and the vegan package v2.6-10 in R v4.3.1 (R Core Team, 2023). We then compared the differences in alpha diversity across various groups using the Wilcoxon rank-sum test or the Kruskal-Wallis H test.

Subsequently, we analyzed beta diversity at both the species and functional levels through Principal Coordinate Analysis (PCoA) and Principal Component Analysis (PCA). The significance of differences in microbial community structure between and within groups was evaluated using Anosim analysis. To further control for potential confounding by BMI, we additionally performed covariate-adjusted Principal Coordinates Analysis (aPCoA) using the method described by Shi et al. (2020). Furthermore, to identify key microbial taxa and functional pathways across different groups, we performed differential abundance analysis utilizing LEfSe (Linear discriminant analysis Effect Size), applying a screening criterion of LDA score > 2 to pinpoint differentially abundant microbes and functional pathways of biological significance. Correlation analyses between differential species and KEGG pathways were performed using Spearman's rank correlation coefficient, and significant associations were visualized as clustered heatmaps. In addition, we classified age-associated microbial taxa and functional pathways into four distinct temporal patterns—continuous increase, continuous decrease, decrease-then-increase, and increase-then-decrease—based on median relative abundance trends across groups. All trend classification and visualizations were implemented in R using the ggplot2 v3.5.2 and ggalluvial v0.12.5 packages (Wickham, 2016; Brunson and QD, 2024). For all differential abundance and correlation analyses involving multiple comparisons, including LEfSe, Spearman correlation, and KEGG pathway enrichment, *p*-values were adjusted using the Benjamini-Hochberg false discovery rate (FDR) method. Results with FDR-adjusted *p*-value < 0.05 were considered statistically significant.

## 3 Results

### 3.1 Demographic characteristics

According to the criteria outlined in the experimental methods section, a total of hundred elderly individuals from Changshou Town participated in this study. The queue comprises thirty two members of the LO group, thirty two members of the MO group, and thirty six members of the YO group. Notably, centenarians were not included in this study to maintain population homogeneity and avoid introducing extreme age-related biological variation. All participants were healthy individuals without any apparent diseases and adhered to a balanced diet consisting of both meat and vegetables. Table 1 presents the demographic characteristics of all participants. As anticipated, age emerged as the most significant distinguishing factor among the three groups. Although this study found statistical differences in BMI among the three groups, BMI was not adjusted for covariates because all participants were healthy elderly and no other confounding factors were identified.

**Table 1:** Comparative analysis of key characteristics among participants across three age groups.

**Characteristics**	**LO (*n* = 32)**	**MO (*n* = 32)**	**YO (*n* = 36)**	***p* value**
Male/female	15/17	16/16	17/19	1.0000
Age(years)	94.16 ± 3.04	81.03 ± 4.18	67.06 ± 3.53	<0.0001
BMI	19.58 ± 2.32	21.23 ± 2.2	21.55 ± 2.16	0.0200
Drinking	2/32	3/32	5/36	1.0000
Smoking	5/32	7/32	9/36	0.6340

### 3.2 Gene diversity and shared gene features across aging groups

**Figure 1:**
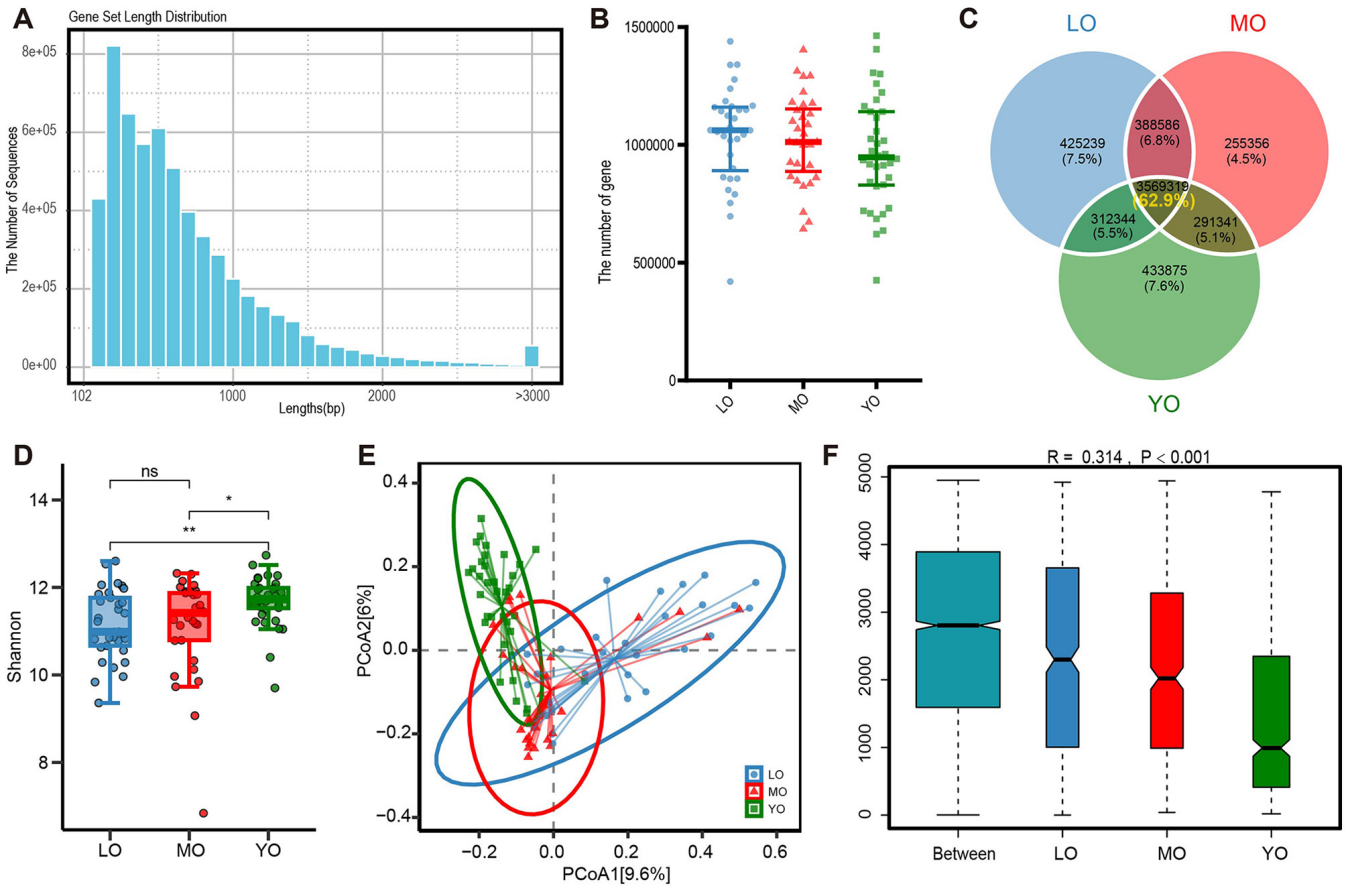
Outcomes of gene annotation analysis derived from metagenomic sequencing of the gut microbiome. **(A)** The histogram illustrates the distribution of lengths within the non-redundant gene set. **(B)** The scatter plot illustrates the differences in the quantity of non-redundant genes among the three groups. **(C)** The Venn diagram depicts the quantity and proportion of both shared and unique genes across the three sample groups. **(D)** The comparison of alpha diversity was conducted using the Shannon index. Each data point corresponds to a sample, while the box plot illustrates the median, quartiles, and outliers. ns, *p-*value > 0.05; *, *p-*value ≤ 0.05; **, *p-*value ≤ 0.01. **(E)** The analysis of β diversity was conducted using the Bray-Curtis distance metric. The principal coordinate analysis (PCoA) plot illustrates the distribution of gene composition across the three sample groups. **(F)** The ANOSIM (Analysis of Similarity) analysis of gut microbiota was conducted using the Bray-Curtis distance metric. The x-axis displays all samples categorized by group, while the y-axis illustrates the rank of the Bray-Curtis distance. The R is between (−1,1): if *R* > 0, the inter-group difference is significant; if R <0, the intra-group difference is greater than the inter-group difference. The reliability of the statistical analysis is represented by the *p*-value, with *p*-value <0.05 denoting statistical significance.

Through metagenomic sequencing, we acquired a total of 10.1 billion raw reads from hundred fecal samples, which corresponded to 1516.72 gigabases (Gbp) of raw sequence data. Following the removal of 6.53% of adapter sequences, low-quality bases, and 0.11% of human host contamination, we ultimately obtained 1415.95 Gbp of clean reads. The number of reads per individual sample varied from 65,641,012 to 132,466,024. To provide further context, the average sequencing depth and quality control information for each group are also summarized in Supplementary Table 1. Utilizing this data, we assembled contigs that were subsequently subjected to gene annotation and redundancy removal, leading to the identification of 5.9 million non-redundant genes, with an average gene sequence length of 683 base pairs (bp) (Figure 1A). The box plot analysis indicated no significant differences in the number of non-redundant genes among the three groups (Figure 1B). The Venn diagram illustrates the distribution of shared and unique genes among the three groups, revealing that the number of shared genes is 3,585,226, which accounts for 62.9% of the total (Figure 1C). Additionally, we evaluated the differences in gene sets among the various groups using the microbial Shannon index and β-diversity measurements based on the Bray-Curtis distance algorithm. The results indicated that gene diversity in the YO group was significantly higher than that in the LO and MO groups, with notable differences in gene composition distribution among the three groups (*p*-value <0.0010) (Figures 1D-F).

### 3.3 Microbial taxonomy annotation

**Figure 2:**
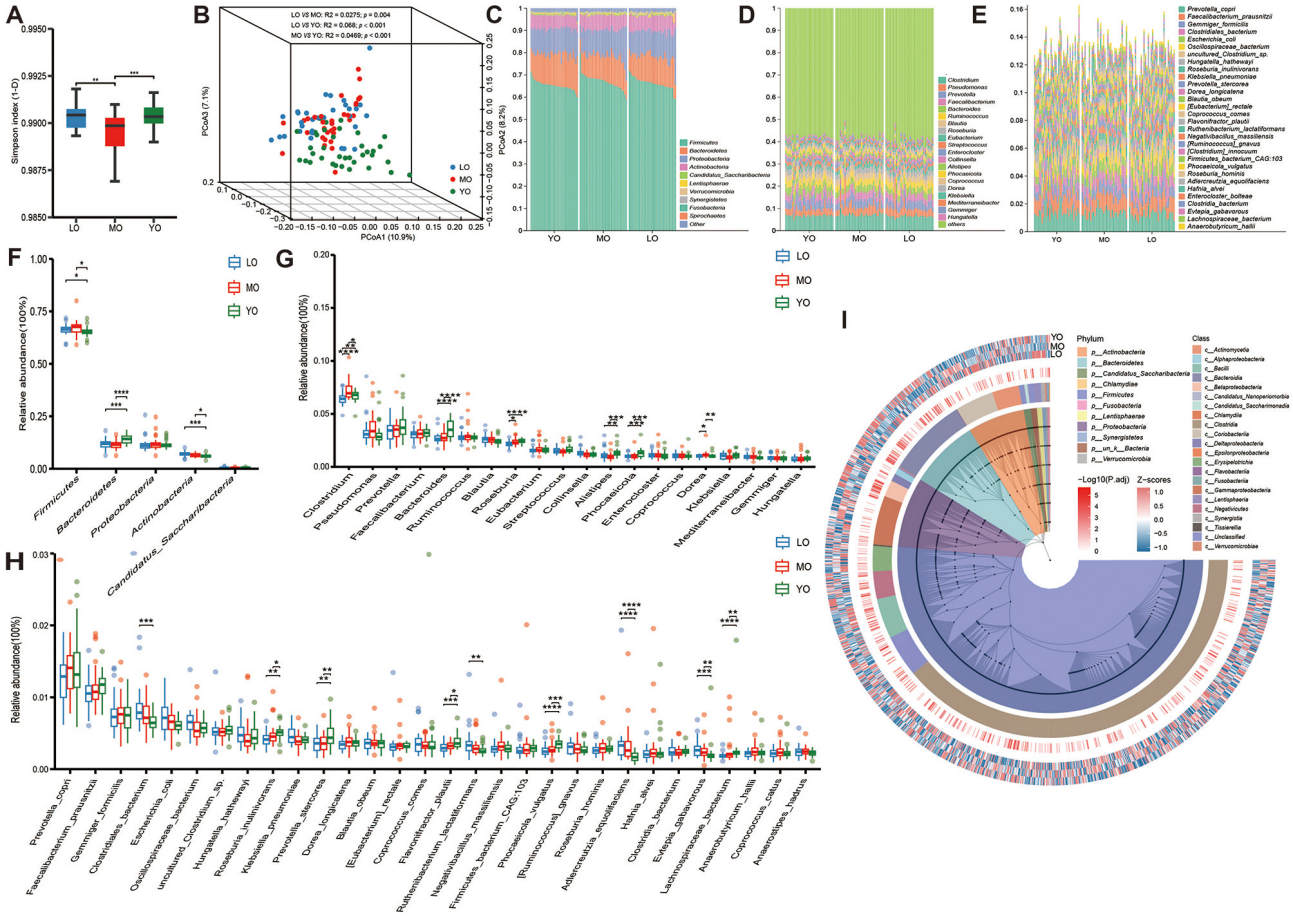
**(A)** The box plot illustrates the α diversity, as measured by the Simpson index, among three distinct groups of gut microbiota. **(B)** The 3D PCoA obtained from the Bray-Curtis distance matrix illustrates the composition of gut microbiota across three groups: LO (blue), MO (red), and YO (green). The analysis highlights the first, second, and third principal components, which collectively account for 10.9%, 8.2%, and 7.1% of the total variance within the dataset, respectively. **(C-E)** The bar charts sequentially display the top 10 phyla, top 20 genera, and top 30 species ranked by their average relative abundance across various taxonomic levels. **(F-H)** The box scatter plots sequentially show the differences in the top 5 phyla, top 20 genera, and top 30 species ranked by average relative abundance across the three groups. Each point represents an individual sample, while the box delineates the median, quartiles, and outliers. **(I)** The species evolutionary circle diagram effectively illustrates the clustering evolution of all species across three distinct groups. The colors in the innermost circle correspond to various phyla levels, while the colors in the second layer denote the different class levels of the species. The gradient in the third layer, transitioning from white to red, represents species that exhibit significant differences among the three groups. Finally, the outermost layer presents a clustered heatmap depicting the average relative abundance of species. *p-value ≤ 0.05; **p-value ≤ 0.01; ***p-value ≤ 0.001; ****p-value ≤ 0.0001.

Based on metagenomic sequencing data, we conducted a detailed analysis of the bacterial component of the gut microbiota. The differences in alpha diversity among the various groups were quantified using the Simpson index. The results indicated that the gut microbiota diversity in the LO and YO groups was significantly greater than that observed in the MO group (Figure 2A). Although no significant differences in alpha diversity were detected between the LO and YO groups, the three-dimensional Principal Coordinates Analysis (PCoA) results, derived from the Bray-Curtis distance algorithm, revealed significant differences in pairwise comparisons, including LO vs. YO (*p-*value <0.0500). In addition, an ANOSIM analysis based on the Bray-Curtis distance confirmed significant differences in gut microbiota structure among the three groups (*R* = 0.128, *p*-value <0.001; Supplementary Figure 1A). This finding suggests the presence of spatial heterogeneity in the gut microbiota structure among the three groups (Figure 2B). After applying aPCoA to adjust for BMI, the clustering patterns and separation among age groups remained largely unchanged, indicating that BMI did not significantly confound the observed gut microbiota differences (Supplementary Figure 1B). Next, we analyzed the gut microbiota at the phylum, genus, and species levels (Figures 2C–E, Supplementary Table 2). The predominant phyla were *Firmicutes, Bacteroidetes, Proteobacteria*, and *Actinobacteria*. Detailed inter-group differences are shown in Figure 2 and Supplementary Table 2. To focus on the most abundant phyla, Figure 2F shows only the top five phyla by average relative abundance. This shift resulted in a significant decrease in the *Firmicutes/Bacteroidetes* ratio in the YO group relative to the other two groups (Supplementary Figure 1C). In our comparative analysis of the top 20 genera by relative abundance, we found that *Clostridium* and *Roseburia* were significantly lower in the LO group than in the MO and YO groups, while *Bacteroides, Alistipes*, and *Phocaeicola* exhibited significantly higher levels in the YO group compared to the MO and LO groups. Furthermore, the levels of *Clostridium* and *Dorea* in the MO group were significantly elevated compared to those in the other two groups (Figure 2G). Further examination at the species level revealed no significant differences in the number of species detected among the three groups. However, a slight increasing trend in species numbers with age was observed (Supplementary Figure 1D). The Venn diagram indicated that the proportion of species shared among the three groups was as high as 85.1%, with the LO group exhibiting a greater variety of unique microbial species compared to the other two groups (Supplementary Figure 1E). Notably, several species such as *Roseburia inulinivorans* and *Prevotella stercorea* were enriched in the YO group, while *Clostridiales bacterium* showed higher abundance in the LO group (Figure 2H, Supplementary Table 2). The phylogenetic tree constructed based on all species illustrated the relative abundance and differential characteristics of species among the different groups (Figure 2I).

### 3.4 Analysis based on taxonomic level

**Figure 3:**
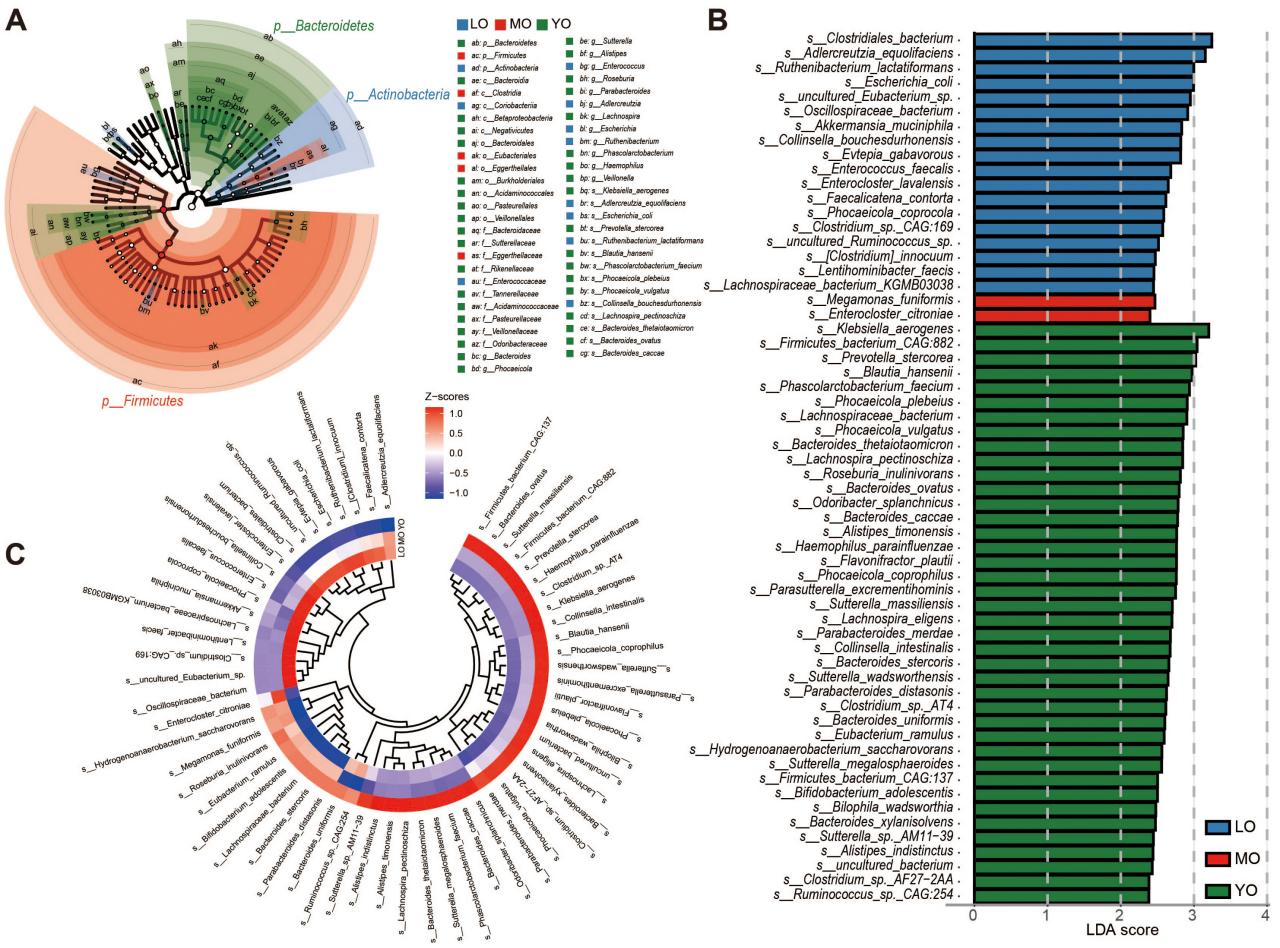
LEfSE analysis and inter-group differential species distribution. **(A)** The phylogenetic tree illustrates the distribution differences of enriched species across various phylum levels in the LO (blue), MO (red), and YO (green) groups. **(B)** The bar chart depicts species that exhibit significant differences among the three groups, determined by effect size (LDA score > 2). The differential species presented in **(A, B)** were identified using the Kruskal-Wallis rank sum test. **(C)** A species clustering evolutionary heatmap was constructed through hierarchical clustering, based on the relative abundance of the previously identified differential species.

Based on species-level LEfSe (Linear discriminant analysis Effect Size) differential analysis, a total of sixty representative differential species were identified among the groups (Supplementary Table 3). These species were detected in samples where the relative abundance exceeded 0.1% in at least 20% of the cases. Overall, the differential bacteria within the LO, MO, and YO groups were predominantly represented by *Actinobacteria, Firmicutes*, and *Bacteroidetes*, respectively, as illustrated in the phylogenetic tree (Figure 3A). Among these species, forty were identified as specific microbial markers in the YO group, followed by the LO group with eighteen species, and the MO group, which contained the fewest species, totaling only 2 (*Megamonas funiformis* and *Enterocloster citroniae*) (Figure 3B). The clustering heatmap further depicted the distribution patterns of these differential species across the various groups (Figure 3C). We further categorized the 60 species into four temporal abundance patterns with aging (Supplementary Table 4, Supplementary Figure 2): (1) Continuous increase pattern (CIP): 13 species, including *Clostridium sp. CAG:169, uncultured Eubacterium sp., Enterocloster lavalensis*, and *Enterococcus faecalis*, were progressively enriched with age, especially in the LO group. (2) Continuous decrease pattern (CDP): 28 species showed a declining trend with age, such as *Megamonas funiformis, Bifidobacterium adolescentis*, and *Bacteroides stercoris*, the latter exhibiting a consistent and significant decline. (3) Decrease-then-increase pattern (DIP): 18 species first declined and then rebounded in the LO group. Notably, 13 of these (e.g., *Prevotella stercorea, Phascolarctobacterium faecium*) were significantly enriched in the YO group. (4) Increase-then-decrease pattern (IDP): Only *Enterocloster citroniae* followed this pattern, with a significant peak in the MO group before returning to YO levels in the LO group.

### 3.5 Analysis based on functional pathways

**Figure 4:**
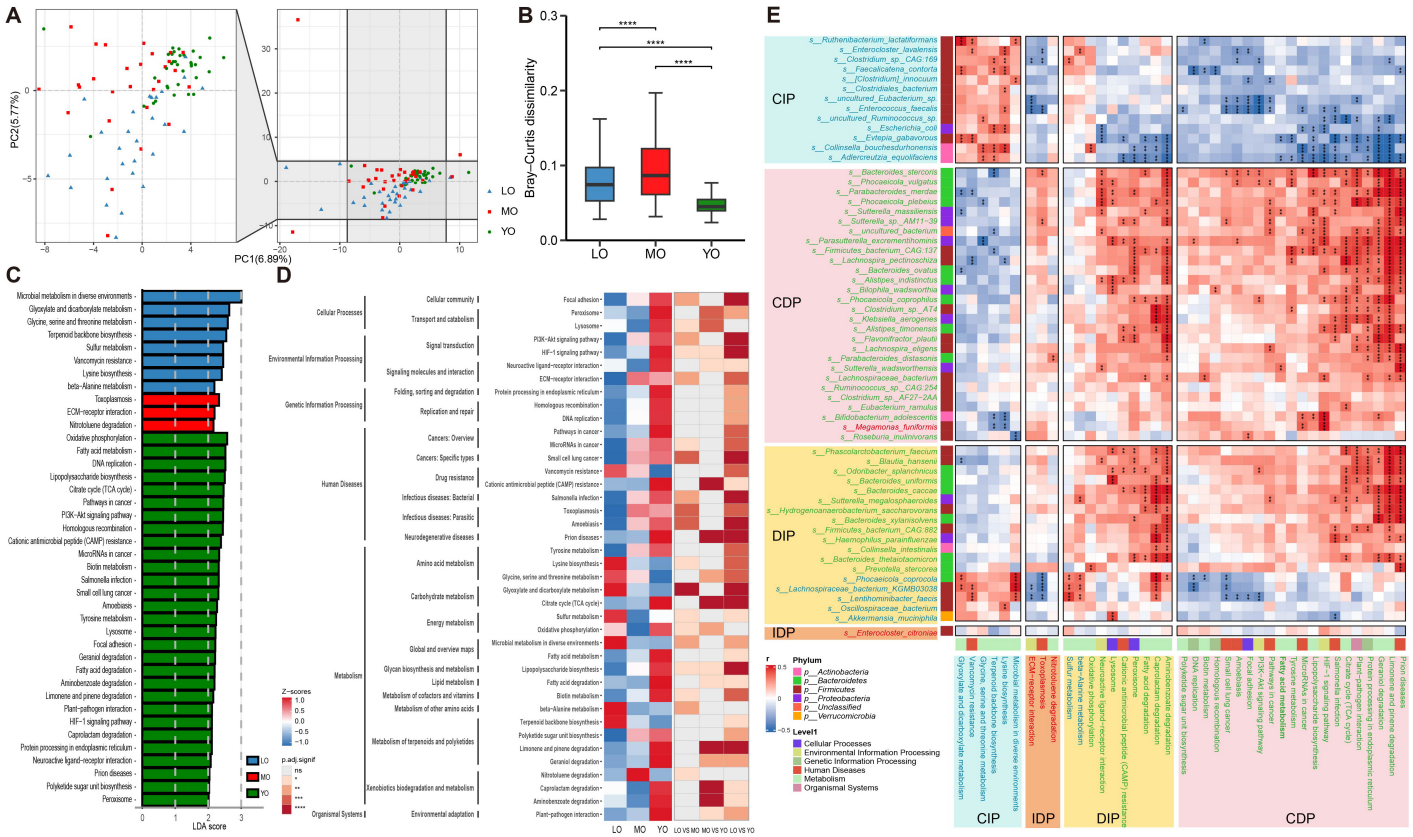
**(A)** The analysis of dimensionality reduction using Principal Component Analysis (PCA) is conducted based on KEGG Level 3 pathways, with the left image presenting a magnified view of the data. **(B)** The box plot illustrates the inter-group differences as determined by the Bray-Curtis dissimilarity distance. **(C)** The bar chart illustrates the KEGG Level 3 pathways that exhibit significant differences among the three groups, as determined by effect size (LDA score > 2). **(D)** A heatmap illustrating the relative abundance distribution of differential pathways identified through KEGG pathway LEfSe analysis across the three groups is presented, alongside a heatmap depicting significant differences observed in pairwise comparisons between the groups. **(E)** A correlation heatmap has been constructed based on the selected differential species and pathways. Distinct font colors denote the species and pathways that are enriched within the corresponding groups. The colors displayed on the left and below the heatmap correspond to the KEGG Level 1 pathway information and phylum-level classification information, respectively. Furthermore, the data is categorized into four distinct trends: CIP, Continuous increase pattern; CDP, Continuous decrease pattern; DIP, Decrease-then-increase pattern; IDP, Increase-then-decrease pattern. **p-value ≤ 0.01; ***p-value ≤ 0.001; ****p-value ≤ 0.0001.

To further elucidate the functional differences in gut microbiota across the three age groups, we conducted a functional annotation of all fecal samples utilizing the KEGG database. A total of three hundred and ninety four pathways were identified at KEGG pathway level 3. Among these, one hundred and sixty six pathways were classified under the KEGG level 1 category of metabolism (42.13%), seventy six pathways were associated with human diseases (19.29%), and seventy four pathways were categorized under organismal systems (2.18%). Additionally, we identified thirty six pathways related to environmental information processing, twenty two pathways associated with genetic information processing, and twenty pathways pertaining to cellular processes (Supplementary Table 5). Principal Component Analysis (PCA) for dimensionality reduction indicated a potential separation trend among the samples from the three groups. Furthermore, an evaluation using the Bray–Curtis dissimilarity index confirmed significant functional differences among the three groups (Figures 4A, B).

To delineate distinct functional pathways among the groups, we conducted LEfSe analysis to pinpoint divergent pathways. We identified 40 pathways significantly associated with metabolism and human diseases across the three groups, as depicted in Figures 4C, D and Supplementary Table 6. Remarkably, more than 70% of these pathways showed a preferential enrichment in the YO group. This enrichment in the YO group may signify a youthful physiological state typified by heightened metabolic activity, although further experimental validation is required to confirm causality.

The clustering heatmap provides a visual representation of the differences in the relative abundance of these pathways among the various groups (Figure 4D). Results from pairwise comparisons indicate that the LO and YO groups exhibit the most significant differences in pathways, while the MO group, which represents an intermediate stage of aging, shows relatively fewer distinctions compared to the other two groups. The 40 age-associated functional pathways were classified into four temporal patterns (Supplementary Table 7, Supplementary Figure 3): (1) CIP: Six pathways increased with age, including lysine biosynthesis, glyoxylate and dicarboxylate metabolism, and microbial metabolism in diverse environments, predominantly enriched in the LO group. (2) CDP: 21 pathways declined with age, notably the TCA cycle, lipopolysaccharide biosynthesis, and HIF-1 signaling pathway, enriched in the YO group. (3) DIP: 10 pathways such as oxidative phosphorylation and lysosome showed a U-shaped trend—decreasing in MO and increasing again in LO. (4) IDP: Three pathways including ECM-receptor interaction and toxoplasmosis peaked in MO then declined in LO.

In addition, we established a connection between the differential species and functions associated with age-related changes, utilizing species function configuration information (Figure 4E). We categorized the species and functions according to four distinct change trends and observed that the alterations in the primary differential functions were consistent with the change trends of the principal differential species, indicating a strong positive correlation. This suggests that the differential changes in these functions are primarily influenced by the variations in these key differential species. Notably, the degradation pathways of caprolactam, geraniol, aminobenzoate, limonene and pinene, as well as fatty acids and prion diseases in the YO group, exhibited a high positive correlation with the majority of the differential species enriched in this group. Among these, *Bacteroides stercoris* stands out as the only species that continuous significantly decreased with age; it was closely associated with the aforementioned pathways and demonstrated a significant positive correlation with lipopolysaccharide biosynthesis (*r* = 0.4910, *p*-value <0.0001) (Figure 4E, Supplementary Table 8). Furthermore, we identified that oxidative phosphorylation pathway, which is intricately linked to mitochondrial function, was significantly positively correlated with *Prevotella stercorea* enriched in the YO group (*r* = 0.3860, *p-*value = 0.0010) and *Collinsella bouchezdurhonensis* enriched in the LO group (*r* = 0.401, *p*-value <0.0010). The pattern of oxidative phosphorylation exhibited an initial decrease followed by an increase, decreasing in the MO group and increasing in the LO group, with no significant difference observed between the LO and YO groups (median LO:YO = 0.0084: 0.0087; *p-*value = 0.5290). Additionally, *Collinsella bouchezdurhonensis* was closely associated with the functions related to glycine, serine, and threonine metabolism enriched in the LO group, along with *Adlercreutzia equolifaciens* and *uncultured Ruminococcus sp*. It is also noteworthy that we discovered significant enrichment of the vancomycin resistance function in the LO group, which was primarily positively correlated with *Lentihominibacter faecis, Lachnospiraceae bacterium* KGMB03038, *Evtepia gabavorous, Ruthenibacterium lactatiformans*, and *Enterocloster lavalensis*, while exhibiting a negative correlation with *Lachnospira pectinoschiza* and *Parabacteroides merdae* enriched in the YO group.

### 3.6 High-quality genome reconstruction reveals key taxa associated with aging

**Figure 5:**
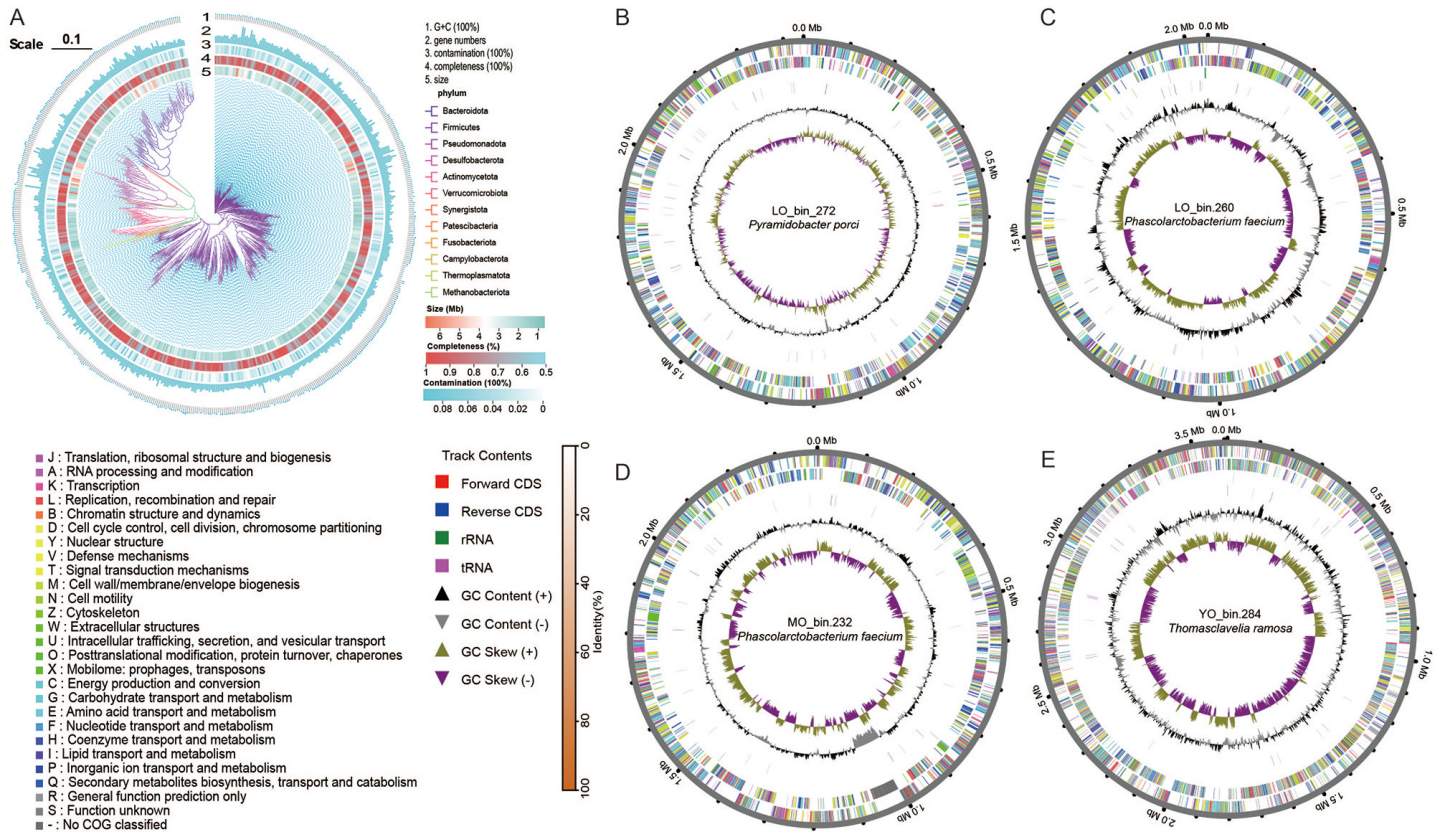
**(A)** A phylogenetic tree based on clustering was constructed using 623 medium to high-quality genomic bins obtained through species binning. The coloration of the various branches corresponds to different phylum levels. Furthermore, the outer ring provides information across five levels, detailing the G + C content (100%), gene counts, contamination rates, completeness, and overall size of the respective genomes. **(B-E)** figures present genomic sketches of species with 100% completeness, derived from the assemblies of the three groups.

Finally, we employed the MetaWRAP pipeline methodology to reconstruct a total of 1,144 medium to high-quality metagenome-assembled genome bins from three groups of fecal metagenomes. Notably, four species were assembled with 100% completeness, and to complement this, Supplementary Table 9 also provides the average genome coverage calculated for each group. Following the removal of redundancy, we obtained a total of 623 non-redundant bins (Supplementary Table 10), which included 238 high-quality bins (completeness ≥90%, contamination rate <5%) and 365 medium-quality bins (completion >50% and contamination <10%). The genome sizes of the high-quality metagenome-assembled genomes (MAGs) varied from 1.12 Mb to 6.74 Mb, with an average size of 2.32 Mb. The N90 lengths ranged from 1.09 to 88.35 kb, while the N50 lengths varied from 1.78 to 254.28 kb, with an average of 22.56 kb. The average G + C content for each bin ranged from 24.9% to 72.4% (Supplementary Table 11). Subsequently, we conducted species classification annotation on the 623 non-redundant bins utilizing the Genome Taxonomy Database (GTDB r214). The phylogenetic tree constructed based on species-level bins illustrated the clustering patterns at the genomic level (Figure 5A). The species were predominantly distributed across the phyla *Firmicutes* (comprising 446 species), *Bacteroidota* (81 species), *Pseudomonadota* (44 species), and *Actinomycetota* (32 species). At the genus level, we identified 16 species within *Prevotella*, 13 species each in *Vescimonas* and *Bacteroides*, and 10 species each in *Enterocloster* and *Alistipes* (Supplementary Table 11). The four bins exhibiting 100% completeness, namely LO_bin.260, LO_bin.272, MO_bin.232, and YO_bin.284, were annotated as *Phascolarctobacterium faecium, Pyramidobacter porci, Phascolarctobacterium faecium*, and *Thomasclavelia ramosa*, respectively. It is noteworthy that LO_bin.260 and MO_bin.232 were annotated as the same species. The genomic map derived from the species genome annotation results is presented in Figures 5B-E.

In addition, we identified the genome of the archaeon *Methanobrevibacter smithii A* (YO_bin.249, LO_bin.17, and MO_bin.193) in all three groups, which is classified within the *Methanobacteriaceae* family. Furthermore, an unidentified archaeon from the *Methanomethylophilaceae* family was detected in the LO group (LO_bin.167). Additionally, there are 17 unidentified species that may be regarded as potential new species, among which LO_bin.42 and LO_bin.368 can be inferred as potential new genera.

## 4 Discussion

While many studies have examined gut microbiota in aging populations and centenarians, findings remain inconsistent due to variation in health status, environment, and methods (Wu et al., 2019; Luan et al., 2020; Mancabelli et al., 2024; Gyriki et al., 2025). The aging process is often accompanied by the emergence of various age-related systemic diseases, which complicates the acquisition of samples from healthy elderly populations. In this study, we recruited hundred healthy elderly individuals from a well-characterized longevity region to reduce confounding factors and enhance internal validity. By analyzing the gut microbiota and functional capacity across different aging stages, we aimed to identify microbial features associated with healthy aging. These insights may inform future microbiome-targeted strategies to support healthy lifespan extension.

The prevailing hypothesis suggests that gut microbiota diversity diminishes with natural aging and is closely associated with frailty in older adults (Ragonnaud and Biragyn, 2021; Donati Zeppa et al., 2022; Wang et al., 2024; Yan et al., 2025). The reduced diversity in the MO group suggests that middle-aged elderly experience a gut microbiota decline, often linked to fewer beneficial bacteria (e.g., *Bifidobacteria*) and more opportunistic pathogens, increasing metabolic and inflammatory disease risks (Dey and Ray Chaudhuri, 2023; Khaledi et al., 2024). In contrast, healthy long-lived individuals tend to maintain a more “youthful” gut microbiota with higher diversity, aligning with studies showing that centenarians resemble younger adults in this aspect, which may help sustain health and longevity (Badal et al., 2020; Wang et al., 2022; Pang et al., 2023). This study provides exploratory evidence for the association between gut microbiome features and different stages of healthy aging. However, due to its cross-sectional design, it cannot establish temporal relationships or infer causality. The observed microbial and functional alterations may be consequences, contributors, or mere correlates of aging. Therefore, these findings should be interpreted as hypothesis-generating, and future longitudinal studies are necessary to confirm the directionality and causal relevance of these associations. Furthermore, our investigation also encompassed the genetic diversity of the gut microbiota. Analyses revealed that the genetic diversity in the YO group was significantly greater than that in the LO and MO groups, a finding we speculate may be linked to a younger and healthier physiological state. This evidence further underscores the significance of gut biodiversity in promoting healthy aging.

In the present study, we investigated the potential associations between variations in differential microbial taxa and functional pathways across different age groups. *Clostridium sp. CAG:169* and *Enterocloster lavalensis*, both known SCFA producers, increased with age and were more abundant in the LO group (Zhou et al., 2017; Grenda et al., 2022). *Enterococcus faecalis* is a gut commensal but may cause infections and shows notable antibiotic resistance (Haas and Blanchard, 2020; García-Solache and Rice, 2019; Shah and Varahan, 2024). In our study, we observed that vancomycin resistance-related pathways were relatively enriched in the LO group, although no direct association with *Enterococcus faecalis* was identified. It is crucial to emphasize that the potential for Horizontal Gene Transfer (HGT) of resistance genes represents a primary mechanism for the dissemination of antibiotic resistance. Conversely, *Bacteroides stercoris* demonstrated a significant decreasing trend with advancing age. Correlation analyses indicated that this species was associated with lipopolysaccharide biosynthesis, consistent with Ni et al. (2023), whose mouse experiments suggested that *B. stercoris* may promote NAFLD progression through LPS and BCAA production. However, its higher abundance in the YO group and recent evidence from Ryu et al. (2024), showing that certain strains can inhibit fat accumulation, imply that the effects of *B. stercoris* may be strain-specific and context-dependent. These contrasting findings highlight the need for further functional studies to clarify its precise role. The YO group showed an enhanced capacity for xenobiotic metabolism, which may reflect complex interactions between aging, environment, and lifestyle factors (Collins and Patterson, 2020; Rampelli et al., 2020; Wu et al., 2022; Jin et al., 2023). However, all observed associations between microbial species and KEGG pathways in this study are correlational. While certain taxa appear enriched alongside specific functional pathways, these do not establish mechanistic causality.

We focused on oxidative phosphorylation (OXPHOS), a key component of oxidative metabolism known to decline with age (Santos et al., 2024; Tkemaladze, 2024). Consistent with this, the YO group showed the highest OXPHOS capacity, while the LO group appeared to mitigate this decline, possibly contributing to longevity. In our investigation, *Prevotella stercorea* was notably enriched in the YO group, consistent with Tett et al. (2021), who observed its decline from adulthood to old age. *P. stercorea* is the second most prevalent species in the *Prevotella* genus, following *P. copri*, and is known to produce SCFAs such as acetate and branched-chain SCFAs like isovalerate, which play important roles in gut health and immune modulation (Hayashi et al., 2007). Although our correlation analysis suggests a potential link between *P. stercorea* and OXPHOS, direct mechanistic evidence is still lacking. Given the marked species and strain variability within the *Prevotella* genus, its net effect on host health may vary with diet and host context. In the LO group, *Collinsella bouchesdurhonensis* showed a positive correlation with OXPHOS capacity. Although it has been reported to be enriched in patients with sickle cell anemia and type 2 diabetes (Delgadinho et al., 2022; Saleem et al., 2022), current evidence is limited and inconsistent, and its role in host metabolism remains unclear. These correlations may reflect indirect or combined effects of multiple species and require further clarification.

Our metagenomic assembly successfully reconstructed four high-quality bins and complete genomes of *Methanobrevibacter smithii A*, now classified as *Candidatus Methanobrevibacter intestini* (Chibani et al., 2022). This methanogenic archaeon is a dominant member of the human gut microbiome and has been reported to increase with age and longevity (Wu et al., 2019; Mohammadzadeh et al., 2025). Recent research by Mohammadzadeh et al. (2024) highlighted its interactions with butyrate-producing bacteria, suggesting that such cohabitation could help mitigate the age-related decline of the Lachnospiraceae family, a key SCFA producer. However, the precise mechanisms by which *M. smithii* contributes to healthy aging remain poorly understood and warrant further investigation. Moreover, our high-quality genome reconstructions provide a valuable foundation for identifying novel taxa and exploring the functional mechanisms that link the gut microbiome to longevity.

This study was conducted in Changshou Town, a region known for longevity. Although these findings may be regionally specific due to geographic and ethnic homogeneity, they provide valuable insight into gut microbiota features of healthy aging. To enhance generalizability, future studies should adopt multi-center cohorts covering broader ethnic, dietary, and environmental backgrounds. In addition, the relatively small sample size is a limitation that future studies should address by including larger, more diverse populations. BMI was adjusted using the aPCoA method, showing minimal impact on gut microbiota patterns despite slight baseline differences due to age-related changes. Detailed clinical parameters and lifestyle data (e.g., diet, medication, physical activity) were not collected to minimize participant burden, as the cohort was relatively homogeneous. Nonetheless, residual confounding cannot be fully excluded. Future larger cohorts with detailed clinical and lifestyle assessments are needed to clarify these influences. Finally, while metagenomic sequencing offers detailed species-level insights, it does not capture microbiome metabolic outputs or host interactions. Integrating metabolomics and transcriptomics, combined with long-term longitudinal studies, will be essential for clarifying causal links between the gut microbiota and healthy aging.

In conclusion, this study provides a comprehensive analysis of the characteristics of the gut microbiota in healthy older adults across different age groups during the latter stages of life. It highlights the changes in species composition and functional trends within the gut microbiota as individuals age. Although there are certain limitations to this research, it lays a foundational framework for future investigations into the role of gut microbiota in the context of aging and health. By utilizing more extensive multi-omics methodologies and broadening population studies, subsequent research has the potential to yield deeper insights, ultimately contributing to the identification of potential microbial targets for future interventions. However, longitudinal and interventional studies are required to confirm whether these associations represent causal mechanisms.

## Data Availability

The original contributions presented in the study are publicly available. This data can be found here: https://www.ncbi.nlm.nih.gov/bioproject/PRJNA1195999, accession number PRJNA1195999.
