# Supplementary material for: Gut microbiome dynamics and functional shifts in healthy aging: insights from a metagenomic study
Source: Front Microbiol. 2025 Sep 18;16:1629811. doi: 10.3389/fmicb.2025.1629811 (PMC12490327; doi:10.3389/fmicb.2025.1629811)
Supplement: Supplementary file 1 [file Supplementary_file_1.docx]

**Figure S1-3**


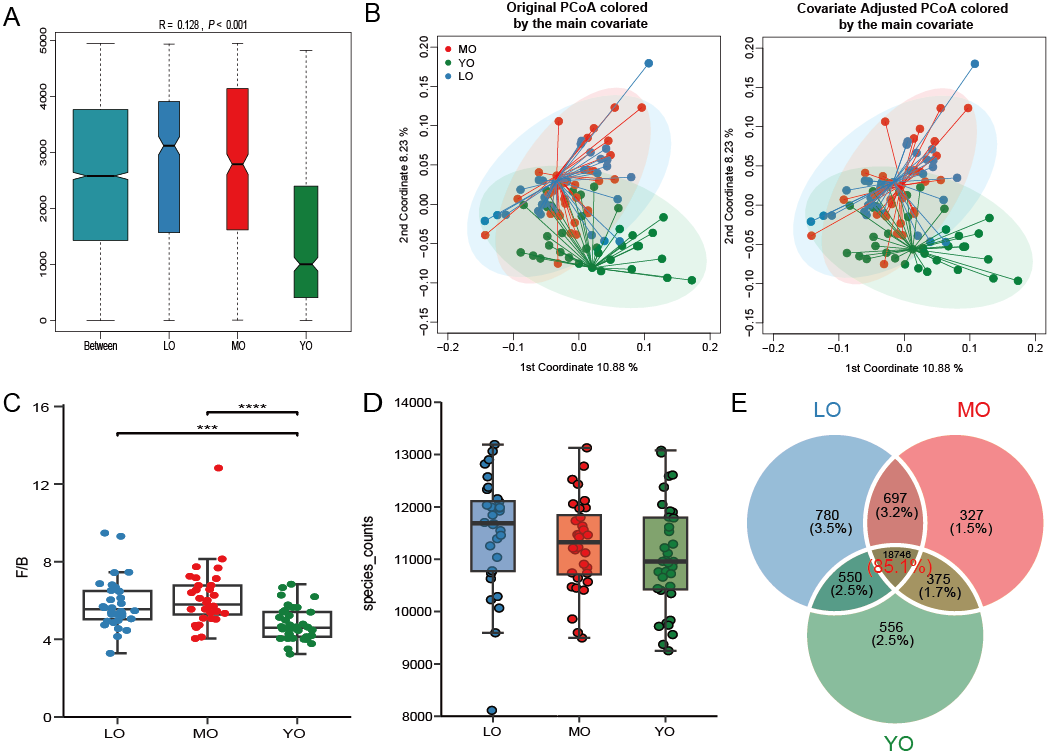


**Figure S1.** (**A**) Analysis of similarity (ANOSIM) based on Bray–Curtis distance showing significant differences in gut microbiota structure among the three age groups (R = 0.128, *p* value < 0.001). (**B**) Principal Coordinates Analysis (PCoA) plots illustrating the gut microbiota structure before (left) and after (right) covariate adjustment for BMI using the aPCoA method. Each point represents a sample, colored by age group (MO: middle-old; YO: young-old; LO: longevity group). Shaded ellipses indicate the 95% confidence intervals for each group. (C) The box plot illustrates the variations in the *Firmicutes/Bacteroidetes* (F/B) ratio of the fecal microbiomes across the three groups. (D)**,** The box plot illustrates the species counts across the three groups, where each point corresponds to an individual sample. The box itself represents the median, quartiles, and outliers of the data. (E)**,** A Venn diagram illustrating microbial species delineates the quantity and proportion of species that are shared and unique among the three groups.


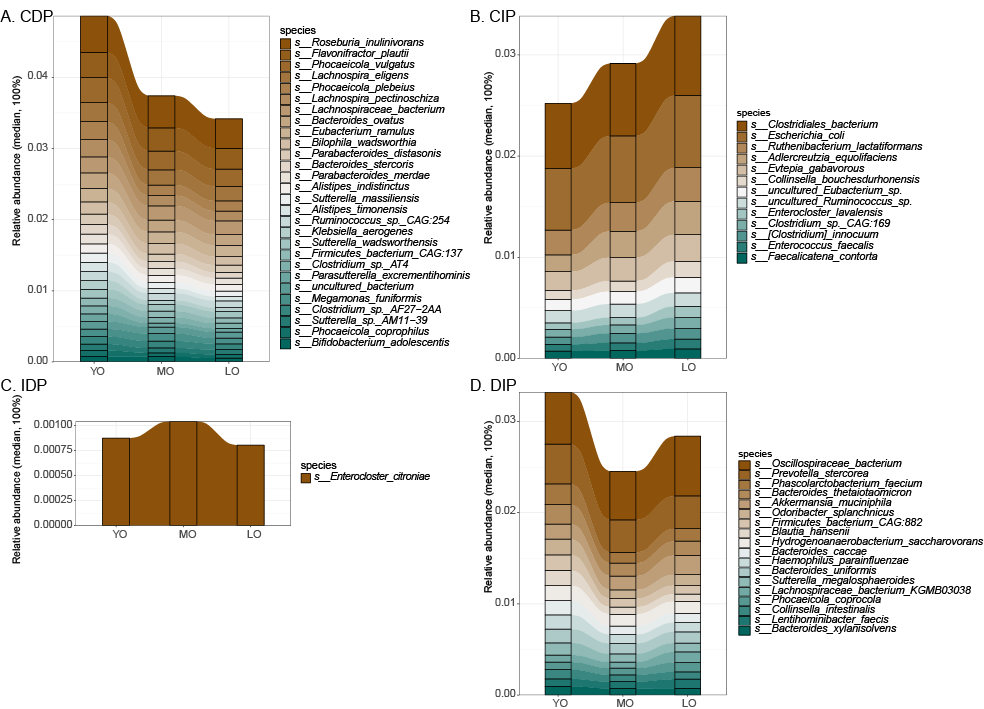


**Figure S2.** Age-related variation patterns in species-level relative abundance, shown as stacked area charts. Age groups are defined as: young-old (YO), middle-old (MO), and long-lived old (LO). (A) Continuous decrease pattern (CDP); (B) Continuous increase pattern (CIP); (C) Increase-then-decrease pattern (IDP); (D) Decrease-then-increase pattern (DIP). The y-axis indicates the median relative abundance (normalized to 100%), and species are color-coded by legend.


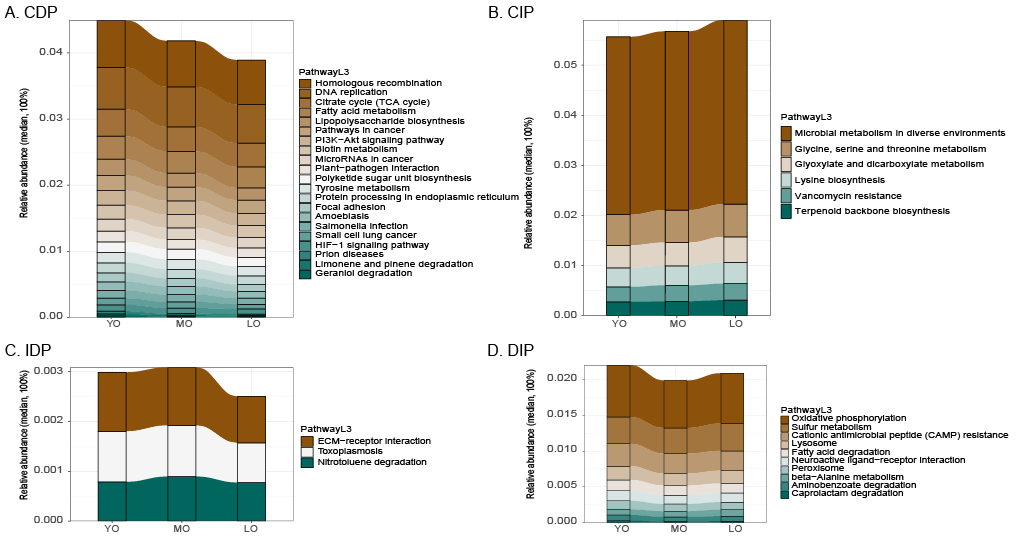


**Figure S3.** Age-related variation patterns in the relative abundance of KEGG level 3 pathways, shown as stacked area charts. Age groups are defined as: young-old (YO), middle-old (MO), and long-lived old (LO. (A) CDP; (B) CIP; (C) IDP; (D) DIP. The y-axis indicates the median relative abundance (normalized to 100%), and pathways are color-coded by legend.
